# Fabrication of Sodium Alginate/Inulin Synbiotic Beads for Protection and Delivery of *Lactobacillus plantarum* in Storage and Simulated Gastrointestinal Conditions

**DOI:** 10.3390/gels12070593

**Published:** 2026-07-03

**Authors:** Weifeng Chen, Xin Li, Richao Hao, Kunpeng Zhao, Jiaxiang Zang, Xiaomin Wei, Wei Xu

**Affiliations:** 1 Institute of Animal Product Quality and Safety Technology, Henan Key Laboratory of Livestock and Poultry Genetic Improvement and Healthy Breeding, Henan University of Animal Husbandry and Economy, Zhengzhou 450046, China; 2 College of Life Science, Dabie Mountain Laboratory, Xinyang Normal University, Xinyang 464000, China

**Keywords:** sodium alginate, inulin, bead, *Lactobacillus plantarum*, stability

## Abstract

In this study, sodium alginate/inulin (SA/IN) composite beads were fabricated using the calcium ion cross-linking method, which encapsulated *Lactobacillus plantarum* to provide it with resistance in a gastrointestinal environment. The results showed that the SA/IN solution functioned as a kind of pseudoplastic fluid, and the storage modulus (G′) and loss modulus (G″) values exhibited a trend of frequency dependence. The diameter, water content, swelling rate, water holding capacity (WHC), and hardness of the composite beads were regulated by IN concentration, with IN making the beads rougher at first and then giving them a more regular shape as the concentration increased. The highest *Lactobacillus plantarum* encapsulation efficiency reached 92.8 ± 4.14%, and SA/IN beads improved the stability of *Lactobacillus plantarum* under 4 °C storage and heat treatment. The quantity of *Lactobacillus plantarum* reached 1.3 ± 0.01 CFU/g, which is close to the quantity observed before digestion. This study confirmed that SA/IN composite beads can serve as a protective carrier of *Lactobacillus plantarum* with prebiotic activity and can be used in functional food ingredients.

## 1. Introduction

Preventing disease through nutrition has become a growing health trend in recent years. Probiotics, as live microorganisms, have been widely recognized for their diverse benefits to the host when provided in adequate amounts [1]. Notably, *Lactobacillus plantarum* is widely used in the food and medical fields, with various confirmed health contributions, such as cholesterol reduction and immune enhancement. Moreover, it promotes physiological functions in different parts of the body, such as the respiratory and urogenital tracts [2,3]. Despite the potential benefits of *Lactobacillus plantarum*, conventional oral administration of probiotics leads to numerous challenges, including reduced activity during their production, transportation, and exposure to the extreme environment of the gastrointestinal tract, and their precise delivery to specific intestinal sites [4].

Therefore, microencapsulation technologies such as extrusion, emulsification, freeze-drying and spray-drying have been applied to overcome these key challenges [5,6]. Microcapsules prepared using different wall materials and preparation processes have different encapsulation effects. Polysaccharides and other macromolecule-based encapsulation systems are commonly developed to improve the activity of probiotics in the gut. This is because most polysaccharides have good acid stability and resistance to digestion [7]. For most polysaccharides, the extrusion gel method is commonly used to encapsulate probiotics due to its ease of operation [8]. Previous studies have employed sodium alginate (SA), an anionic polysaccharide with 1,4-linked-dmannuronic acid and α-l-guluronic acid residues that forms an “egg-box” gel through cross-linking with calcium ions [9,10]. SA beads do not dissolve under low-pH conditions, which makes them an ideal carrier for transporting acid-sensitive bioactive components. However, the large porous structure of SA beads makes them prone to the absorption of acids, enzymes, and other substances, which may exert stress on the encapsulated probiotics [11].

The use of composite SA beads formulated with other macromolecules was found to mitigate this disadvantage [12]. *Lactobacillus plantarum* was enclosed in three different kinds of wall materials to create calcium pectin beads, chitosan–calcium pectin beads, and SA–pectin–whey beads and it was found that SA composite beads demonstrated superior tolerance, improved release properties in simulated gastrointestinal fluids, and exceptional stability during the storage period [13]. In an in vitro digestion study [14], the authors used a water–oil–water emulsion combined with SA/carboxymethyl chitosan hydrogel shells to encapsulate *L*. *rhamnosus 76* for intestinal targeted delivery. The composite SA beads showed excellent pH responsiveness and good thermal stability, and the survival rate reached 90.69 ± 0.04%. Other macromolecules, such as gum arabic, κ-carrageenan, gelatin, low-methoxyl pectin, konjac glucomannan, low-acyl gellan gum, and xanthan gum, have been combined with SA to prepare composite beads to protect *Limosilactobacillus fermentum*, achieving encapsulation efficiency of 80% to 100% and survival rates of up to 8.1 log CFU/g [15,16,17].

Inulin (IN), a natural fructan polysaccharide, is primarily composed of β-(2,1)-linked D-fructofuranose units [18,19]. It is a soluble prebiotic dietary fiber that can enhance digestive health by modulating the gut microbiota, and can be fermented by *Lactobacillus* and *Bifidobacterium*, producing short-chain fatty acids, thereby promoting immune ability, metabolic health, and overall wellness [20,21,22]. IN extracted from burdock root was incorporated into alginate/chitosan hydrogel beads for probiotic encapsulation, and it was found that it formed dense bead structures that significantly enhanced encapsulation efficiency (~88%) and improved probiotic viability (*>*6 log CFU/g) [23]. In addition, the efficacy of the SA/IN system has also been studied for loading chokeberry (*Aronia melanocarpa* L.) extract [24].

As a commonly used wall material with beneficial activity for gut the microbiota, IN has rarely been studied in combination with SA for loading *Lactobacillus plantarum*. The effect of IN on *Lactobacillus plantarum* encapsulation efficiency is unclear, and currently, there is not enough information about the effects of SA/IN composite beads on the storage and digestive stability of *Lactobacillus plantarum*. Therefore, in the current work, SA/IN composite beads were prepared using the calcium cross-linking method, which were used to entrap *Lactobacillus plantarum* under adverse environmental conditions. The composite beads were further characterized, and we evaluated their encapsulation and delivery of *Lactobacillus plantarum* in low-temperature, heat treatment, and gastrointestinal conditions. The results not only demonstrate that SA/IN composite beads enhance the survival and stability of probiotics but also provide new insight into their protective potential in the functional food and medical fields, for example, as beverage nutrient enhancers and gastrointestinal oral regulators.

## 2. Results and Discussion

### 2.1. *Flow Sweep* of SA/IN Solutions

**Figure 1:**
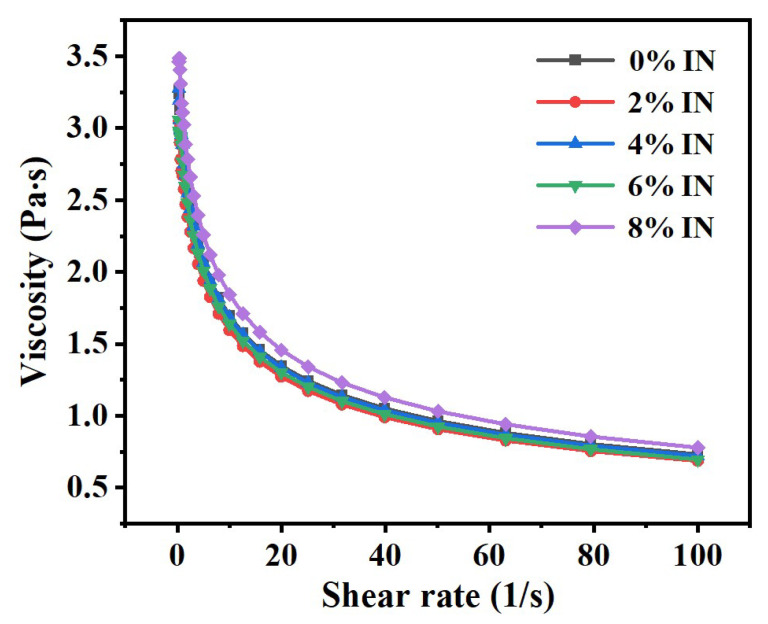
Viscosity as a function of shear rate of SA/IN solution.

As the shear rate increased, the viscosity of the SA/IN solutions significantly decreased (Figure 1). At a low shear rate, the molecules in the SA/IN system were arranged in an orderly manner, and there were strong electrostatic forces that made the internal structure of the system relatively stable and resulted in higher viscosity. The response of the system to the external shear stress was relatively minimal. However, when the shear rate increased, the molecular chain structure within the composite system was disrupted. The entanglement forces between SA and IN molecules, such as hydrogen bonds and van der Waals forces, weakened at high shear rates, and as the concentration of IN increased, so too did the viscosity of the SA/IN composite solutions. On the one hand, IN contributed to the viscosity of the entire system; the higher the concentration, the greater the contribution rate became. On the other hand, the combination of hydrophobic interactions and hydrogen bonds increased the viscosity of the SA/IN system. The results of the Power law model showed that the non-Newtonian index n of the SA/IN solution was less than 1, indicating that the system was a pseudoplastic fluid (Table 1). Moreover, as the IN concentration increased, the K value showed an increasing trend, consistent with the results shown in Figure 1.

### 2.2. Frequency Sweep of SA/IN Solutions

**Figure 2:**
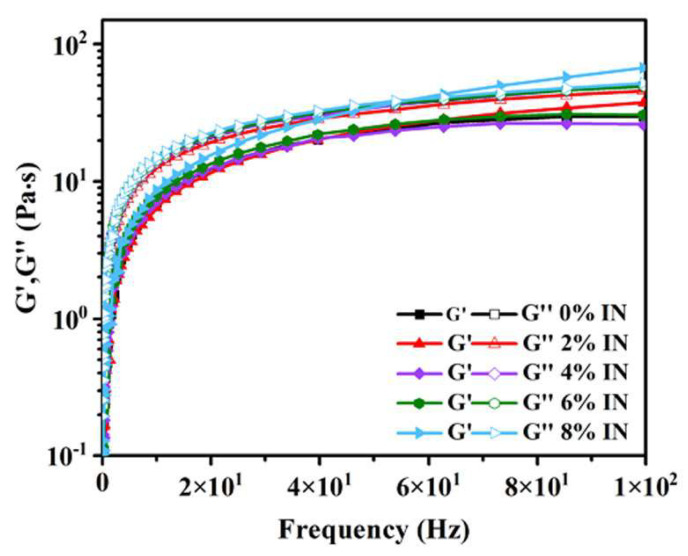
Frequency sweeps of SA/IN solution.

The storage modulus (G′) and loss modulus (G″) are typically used to characterize the viscoelasticity and strength of gel networks. Figure 2 shows the changes in G′ and G″ of the SA/IN composite system during frequency sweep, with both exhibiting frequency dependence, indicating that the temporary network was formed through crosslinking and aggregation. In the low-frequency range, G″ > G′, representing the dominant liquid characteristic. As the frequency increased, G′ gradually increased and became greater than G″, which was attributed to the entanglement network between SA and IN. Briefly, there was enough time for entanglement to occur at low frequencies, while at higher frequencies, the oscillation rate exceeded the duration of molecular entanglements. This phenomenon is similar to that observed in many other polysaccharide or protein systems, such as sodium caseinate/carrageenan [25]. The crossover of G′ and G″ confirmed that different IN concentrations exerted a significant impact on the temporary network structure of the SA.

### 2.3. Diameter and Water Holding Capacity of SA/IN Composite Beads

**Figure 3:**
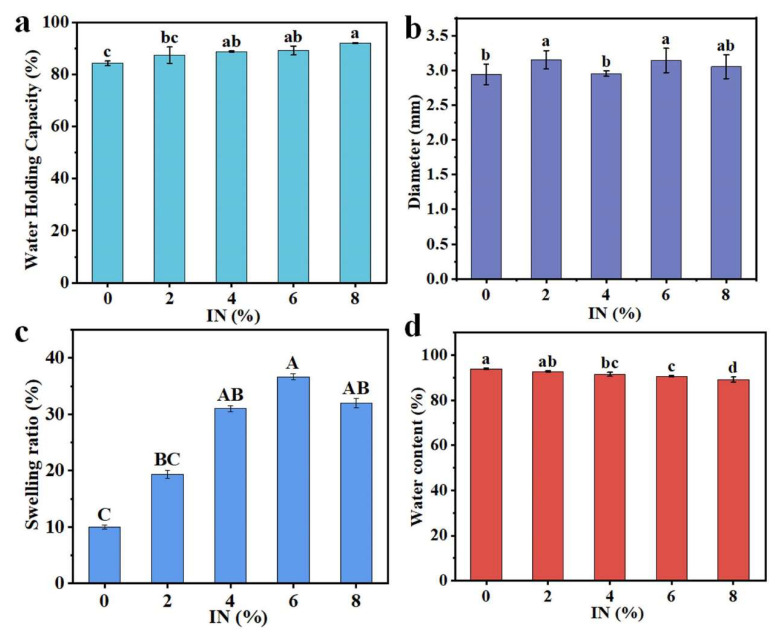
Water holding capacity (**a**), diameter (**b**), swelling ratio (**c**), and water content (**d**) of SA/IN composite beads. Different letters in the same column indicate significant differences (*p* < 0.05).

Figure 3a shows that the diameter of SA/IN composite beads significantly increased as IN concentration increased. The diameter of SA beads was 0.843 ± 0.009 mm, which increased to 0.888 ± 0.001 mm when 4% IN was added to produce SA/IN composite beads. This primarily resulted from the fact that increasing IN enhanced the water absorption and gelation properties of the composite gel. However, when the IN concentration was further increased, the rate of increase in diameter slowed down, indicating that in SA/IN composite beads, SA was the primary contributor to gelation. The water holding capacity (WHC) of SA/IN composite beads upon adding IN exhibited a similar trend to diameter, increasing from 2.94 ± 0.15% to 3.14 ± 0.17% as the IN concentration increased to 6% (Figure 3b). However, at concentrations of 6% and 8%, the WHC did not show any significant changes, and all composite beads exhibited good water retention ability. These results indicate that WHC is impacted not only by the concentration of IN, but also by the density of the gel network. A high concentration of IN may increase the porosity of the gel network and reduce its ability to combine with water through capillary forces [26,27].

### 2.4. Water Content and Swelling Rate Analysis of SA/IN Composite Beads

Water content and swelling rate were found to indirectly reflect the internal molecular interactions within SA/IN composite beads. As shown in Figure 3a, IN exerted different and significant effects on swelling rate and water content. When no inulin was added, the swelling rate of SA beads was only 10 ± 0.34%; this increased to 31 ± 0.56% when 4% was inulin added, and reached a maximum of 36.7 ± 0.58% when inulin was added at 6%. However, a further increase in inulin concentration led to a slight decrease in the swelling rate. This significant improvement in swelling rate might be attributed to the high water-absorbing capacity of inulin. Meanwhile, the water content of SA/IN composite beads decreased slightly with increasing inulin content, and showed a relatively close dependence on IN concentration. This was because SA had higher water-retention ability compared to IN, and excessive addition of IN might disrupt the network structure of SA/IN composite beads.

### 2.5. Hardness and FTIR Analysis of SA/IN Composite Beads

**Figure 4:**
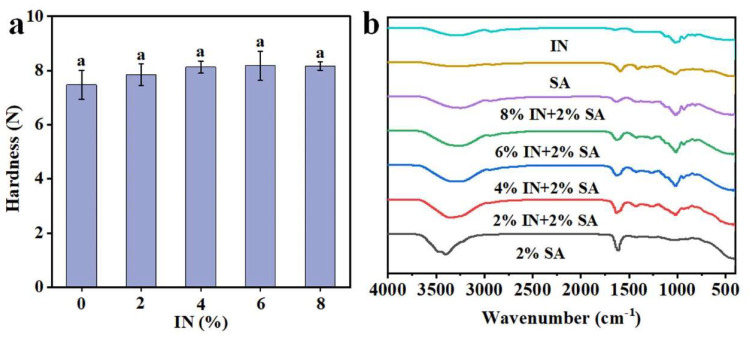
Hardness (**a**) and FTIR analysis (**b**) of SA/IN composite beads. Different letters in the same column indicate significant differences (*p* < 0.05).

According to Figure 4a, as the concentration of IN increased to 8%, the hardness of the composite gel continuously rose from 7.5 N to 8.2 N. Specifically, within the range of 0–4%, the hardness increased slowly, while at 6–8%, the increase slowed down and approached a plateau (8.2 N). This indicated that IN enhanced the mechanical properties of the gel, but there was a saturation effect. Overall, IN had no significant effect on the hardness of the SA/IN composite beads. This was primarily because IN acted as a second network component, which could interpenetrate the primary SA gel network and increase the network crosslinking density only through weaker forces, such as hydrogen bonds and hydrophobic interactions. At low concentrations (0–4%), IN molecules effectively bridged the network structure, and the enhancement was obvious, while concentrations exceeding 6% led to local phase separation; this is consistent with the results shown in Figure 3. The FTIR spectra of the SA/IN composite beads are displayed in Figure 4b. The characteristic peaks of SA at 1030 cm^−1^ and 1412 cm^−1^ resulted from C-O-C stretching vibration and COO-asymmetric stretching vibration, respectively. The peaks of SA/IN composite beads around 3110–3700 were attributed to O-H stretching vibration, which indicated that there were hydrogen bonds between SA and IN.

### 2.6. Morphology of Lyophilized SA/IN Composite Beads

**Figure 5:**
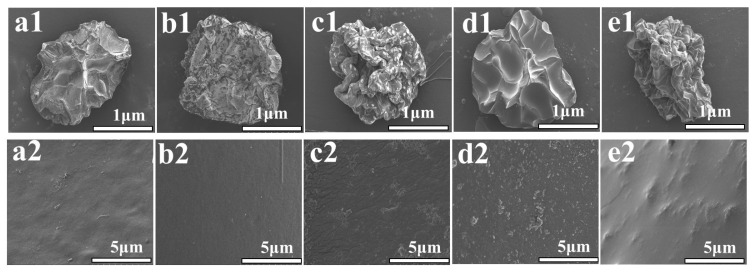
Scanning electron microscopy images of SA/IN composite beads with different IN concentrations ((**a**)—0, (**b**)—2%, (**c**)—4%, (**d**)—6%, and (**e**)—8%; 1 and 2 indicate different magnification factors).

Figure 5 indicates that the morphological characteristics of all SA/IN composite beads were significantly changed when different concentrations of IN were added. It is well known that after freeze-drying, polysaccharide gel becomes wrinkled with a more irregular shape and a heterogeneous surface. The addition of IN gave the beads a regular shape at first, but they became rougher at higher concentrations (Figure S1). In addition, we noted that the SA/IN composite beads underwent microphase separation at high concentrations of IN. The shrinking and shriveling of SA composite beads was primarily attributed to their porous structures and water leakage after lyophilization destabilized the gel network of alginate. Similar morphological observations of lyophilized alginate beads have previously been reported. Nevertheless, the addition of IN could be quite beneficial to overcome the collapse of polymeric gel networks after lyophilization. The results described above illustrate that SA/IN composite beads could be used as an effective carrier for encapsulating *Lactobacillus plantarum*.

### 2.7. *Encapsulation of* Lactobacillus plantarum Using SA/IN Composite Beads

**Figure 6:**
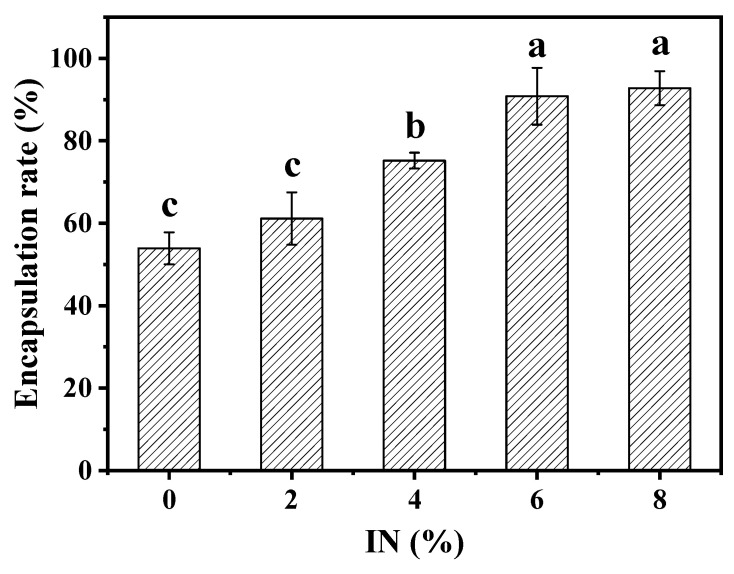
Encapsulation rate of *Lactobacillus plantarum*-loaded SA/IN composite beads. Different letters in the same column indicate significant differences (*p* < 0.05).

Figure 6 shows that the concentration of IN had a significant impact on the *Lactobacillus plantarum* encapsulation rate. The encapsulation rate of *Lactobacillus plantarum*-loaded SA beads was 53.9 ± 3.87%, but in composite beads, with increasing IN concentration, the encapsulation rate gradually increased, reaching the maximum value at an IN concentration of 8%. The highest *Lactobacillus plantarum* encapsulation efficiency was 92.8 ± 4.14%. This may be because the strongest interaction occurred between IN and SA at this concentration; IN was well filled in the SA network gel, and a relatively dense gel network structure was formed, further reducing the leakage of *Lactobacillus plantarum*. It could also be connected to the particle size of SA/IN composite beads; the increase in IN concentration could increase the diameter of the beads, further increasing the *Lactobacillus plantarum* encapsulation rate. Norsyazwani Solehah Norudin et al. also investigated the relationship between encapsulation efficiency and inulin concentration in inulin-based hydrogels, and found that there was a corresponding increase in the protein encapsulation rate as IN concentration increased from 5% to 15%. These results were consistent with the phenomenon observed in this study whereby the presence of inulin improved the capacity to encapsulate and retain greater amounts of protein or probiotics [28].

### 2.8. Stablility *of* Lactobacillus plantarum Loaded into SA/IN Composite Beads During Storage and Heat Treatment

**Figure 7:**
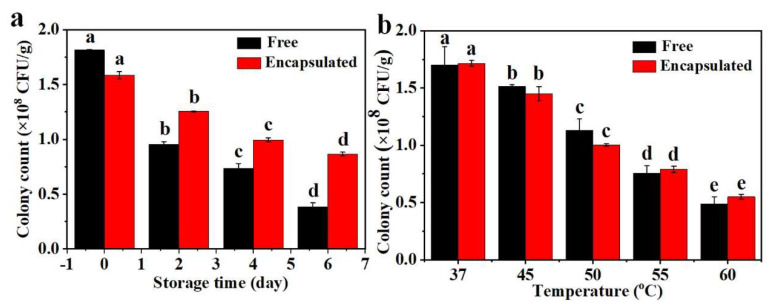
The stability of *Lactobacillus plantarum* during storage (**a**) and temperature stress (**b**) with and without bead loading. Different letters in the same column indicate significant differences (*p* < 0.05).

As shown in Figure 7a, storage time significantly affected the survival rate of *Lactobacillus plantarum*, and longer storage periods resulted in lower survival rates. After 7 days of storage, the number of viable free *Lactobacillus plantarum* cells decreased from the initial 1.82 × 10^8^ CFU/g to 0.39 × 10^8^ CFU/g. Similarly, the *Lactobacillus plantarum* encapsulated within SA/IN composite beads showed a trend of declining free *Lactobacillus plantarum* over time. The viable count remained at 0.87 × 10^8^ CFU/g after 7 days of storage, with an initial level of 1.5 × 10^8^ CFU/g. It demonstrated a twofold increase compared to the free form, indicating that SA/IN composite beads provided effective protection for *Lactobacillus plantarum* during low-temperature storage, and significantly improved its survival rate during long storage periods.

The heat resistance of *Lactobacillus plantarum* was evaluated before and after encapsulation under different heat treatment conditions (Figure 7b). Temperature is an important factor affecting the survival rate of probiotics, and previous studies have suggested that its impact on the survival rate of encapsulated probiotics depends on the duration of contact and the level of temperature [29]. In the present study, the survival rate of probiotics showed a significant dependence on temperature. At lower temperatures (<50 °C), free *Lactobacillus plantarum* survival was high. This was primarily due to the physical effect of the SA/IN composite beads, which acted as a barrier to the circulation of nutrients without affecting free *Lactobacillus plantarum*. When the temperature was above 50 °C, the SA/IN composite beads exerted a better protective effect on Lactobacillus plantarum. At a heat treatment temperature was 55 °C, the survival rate was 0.75 ± 0.06%. The survival rate for the *Lactobacillus plantarum* encapsulated in SA/IN composite beads was 0.79 ± 0.03%. However, when the treatment temperature was raised to 60 °C, the protection rate of SA/IN composite beads was 0.55 ± 0.02%, and it was 0.49 ± 0.06% for free *Lactobacillus plantarum*. This indicates that SA/IN composite beads can effectively protect *Lactobacillus plantarum* at higher temperatures. A possible reason for this phenomenon is that SA and IN exhibited prebiotic activity that facilitated the growth of *Lactobacillus plantarum*. Additionally, the core–shell internal bead structure also improved *Lactobacillus plantarum*’s ability to withstand adverse conditions. It has been reported that IN acts as a multifunctional prebiotic to stimulate gut modulation and provide systemic health benefits. As a soluble prebiotic dietary fiber derived from plants, IN can easily be fermented by *Bifidobacterium* and *Lactobacillus* species, resulting in the production of short-chain fatty acids [30].

### 2.9. Survival of Encapsulated Lactobacillus plantarum in Simulated Gastric Fluid

**Figure 8:**
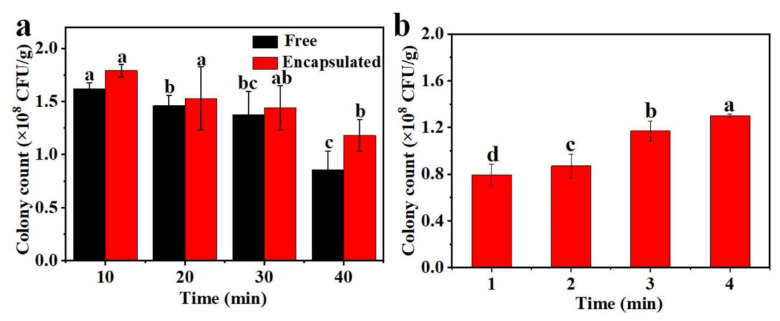
Survival rate of *Lactobacillus plantarum* in simulated gastric (**a**) and intestinal (**b**) fluid. Different letters in the same column indicate significant differences (*p* < 0.05).

Due to the extremely acidic environmental conditions, the viable count of *Lactobacillus plantarum* significantly decreased at the beginning of the simulated gastrointestinal digestion process (Figure 8a). The rate of decline increased over time, with the viable count dropping by half within 40 min, from 1.62 ± 0.06 CFU/g to 0.86 ± 1.17 CFU/g. Compared with the free *Lactobacillus plantarum*, the viable count of *Lactobacillus plantarum* encapsulated by SA/IN composite beads exhibited a similar declining trend. However, its viable count was higher than that of the free *Lactobacillus plantarum* at the same stage of gastric digestion. In the first 20 min, the probiotics embedded on the surface were rapidly released into the simulated gastric fluid, resulting in a significant decrease in bacterial count.

Due to the stable gel structure under acidic conditions, SA/IN composite beads were unable to disrupt the internal structure and cause massive release of *Lactobacillus plantarum*. The loss of encapsulated bacteria from SA/IN composite beads occurred gradually as gastric digestion time increased. It is well known that maintaining a certain number of viable probiotics during the intestinal stage of digestion is important for ensuring beneficial physiological activity. The viable count of free *Lactobacillus plantarum* was almost negligible after gastrointestinal digestion, while in the SA/IN composite beads, *Lactobacillus plantarum* was not only abundant but also significantly increased over time. After 4 h of intestinal fluid digestion, the *Lactobacillus plantarum* count reached 1.3 ± 0.01 CFU/g, which was basically the same as the count before digestion. This was primarily because the three-dimensional network formed by SA and IN prevented the inhibitory effect of the adverse gastrointestinal environment on *Lactobacillus plantarum*. On the other hand, the prebiotic SA and IN might be helpful for promoting growth of *Lactobacillus plantarum* [31].

### 2.10. SEM Observation of Lactobacillus plantarum-Loaded Beads After Gastrointestinal Digestion

**Figure 9:**
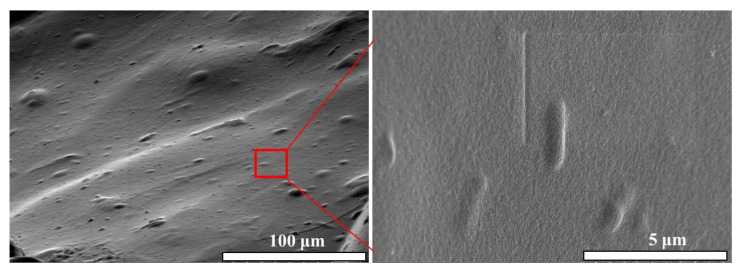
SEM observation of *Lactobacillus plantarum* after gastric digestion.

SEM of *Lactobacillus plantarum* was conducted after gastric digestion to more accurately assess its structure (Figure 9). It was noted that the SA/IN composite beads displayed heterogeneous and intact microstructures, which confirmed that the structure of SA/IN composite beads crosslinked with Ca^2+^ could enable them to survive in the digestive environment. Compared with their microstructure before gastric digestion, the SA/IN composite beads exhibited much more phase separation. Nevertheless, the SA/IN composite beads contained a large number of *Lactobacillus plantarum* cells, with most of them embedded in the beads, rendering them resistant to adverse environmental conditions. The enlarged image further confirms that *Lactobacillus plantarum* displayed a normal physical appearance, and the SEM results illustrate that *Lactobacillus plantarum* was able to develop a normal appearance during gastric digestion. This observation confirms that SA/IN composite beads can not only serve as a protective carrier for *Lactobacillus plantarum* but also exhibit prebiotic activity.

## 3. Conclusions

In this study, SA/IN composite beads were prepared through calcium cross-linking and used to encapsulate *Lactobacillus plantarum*, in order to confer resistance under in vitro gastrointestinal conditions. The SA/IN solution behaved like a pseudoplastic fluid, where G′ and G″ exhibited frequency dependence. When 4% IN was added, the diameter and swelling rate of SA/IN composite beads increased to 0.888 ± 0.001 mm and 31 ± 0.56%, respectively, and WHC increased from 2.94 ± 0.15% to 3.14 ± 0.17% as the IN concentration increased to 6%. IN gave the beads a regular shape at first but made them rougher at high concentrations. The highest *Lactobacillus plantarum* encapsulation efficiency reached 92.8 ± 4.14%. SA/IN beads enhanced the storage stability of *Lactobacillus plantarum* at 4 °C and under heat treatment, especially at high temperatures (<50 °C). After digestion, the number of *Lactobacillus plantarum* cells reached 1.3 ± 0.01 CFU/g, which was similar to the initial number before digestion. The SA/IN composite beads demonstrated efficacy as a protective carrier for *Lactobacillus plantarum* and exhibited prebiotic activity, and can thus be applied in the functional food and medical fields.

## 4. Materials and Methods

### 4.1. Materials

Sodium alginate (SA, M_W_ 1.2 × 10^5^–2.0 × 10^6^ Da) was purchased from Sinopharm Chemical Reagent Co., Ltd. (Shanghai, China); native inulin (IN, DP 2-60) was obtained from the Cosucra Company (Warcoing, Belgium); *Lactobacillus plantarum* was separated from pickles and provided by Henan Key Laboratory of Innovative Utilization of Unconventional Feed Resources; the medium and other chemicals were reagent-grade and purchased from Sinopharm Chemical Reagent Co., Ltd. (China); and the water used in the experiments was ultrapure water prepared using a Milli-Q water system (Millipore, Billerica, MA, USA).

### 4.2. Rheological Properties of SA/IN Solution

The SA solution (2 wt%) was prepared by stirring it for 5 h at room temperature. Solutions with different concentrations of IN (0 wt%, 2 wt%, 4 wt%, 6 wt%, and 8 wt%) were also prepared at room temperature. The SA/IN solutions were mixed with equal volume of SA and IN, and their rheological properties were measured using a DHR-2 rheometer with a 40 mm diameter aluminum plate. Before the test, a linear viscoelastic zone was identified. The shear rate experiment was conducted with the shear rates increasing from 0.1 s^−1^ to 100 s^−1^ at 25 °C. The frequency sweeps were performed in the range of 0.1 Hz to 100 Hz with 0.1% strain. Finally, the strain sweep was tested at 1 Hz, with the strains increasing from 0.01% to 100%.

### 4.3. Preparation of SA/IN Composite Beads

SA/IN composite beads were prepared using our previously reported method with minor modifications [32,33]. The SA/IN blend solutions (10 mL) were collected using a 10 mL syringe, and then dripped into CaCl_2_ (2 wt%) solution with a needle of 0.5 mm diameter, with the distance between the needle tip and CaCl_2_ solution fixed at 15 cm. The composite beads were aged for additional 30 min, and the aged beads with different IN concentrations were collected for further experimentation.

### 4.4. Diameter and Water Holding Capacity Measurements of SA/IN Composite Beads

A micrometer was used to measure the diameter of 10 SA/IN composite beads randomly selected from each group. Water holding capacity (WHC) was measured using a previously reported method with minor modifications [34,35]. SA/IN composite beads were loaded into centrifuge tubes and centrifuged with at 4000 rpm for 20 min at 20 °C. The WHC was calculated according to the following formula.

(1) $$WHC(\%)=\frac{W_{1}}{W_{0}}\times 100$$

where *W*_0_ is the total weight (g) of the SA/IN composite beads before centrifugation, and *W*_1_ is their weight (g) after centrifugation.

### 4.5. Water Content and Swelling Rate Measurement of SA/IN Composite Beads

The water content and swelling rate of SA/IN composite beads were determined using the gravimetric method [36]. Fresh SA/IN composite beads (1 g) were placed in an oven for drying for 24 h at 40 °C, and the constant weight was used to indicate moisture content after drying. To determine the swelling rate, SA/IN composite beads were also dried for 24 h in an oven at 40 °C. After taking them out, their masses were measured and recorded as the initial mass (*m*_1_). Then, they were placed in deionized water at room temperature for 24 h to fully swell. After filtration and separation, the surface moisture of the microspheres was dried with filter paper, and their masses were measured again and recorded as the swollen mass (*m*_2_). The swelling rate of SA/IN composite beads was calculated according to the following formula:

(2) $$\mathrm{Swelling}\;\mathrm{rate}\;(\%)\;\frac{m_{2}-m_{1}}{m_{1}}\times 100$$

### 4.6. Hardness Measurement of SA/IN Composite Beads

The hardness of SA/IN composite beads was measured using a texture analyzer (TMS-Pro 3000, FTC, Virginia, VA, USA) equipped with a P75 probe. All composite beads were compressed to 70% with a speed of 1 mm/s to record the hardness.

### 4.7. *Fourier Transform Infrared (FTIR) Spectroscopy of* SA/IN Composite Beads

First, the SA/IN composite beads were dried using a vacuum freeze-dryer. The freeze-dried composite beads, SA, and IN were mixed with KBr, and tested using an FTIR spectrometer (Perkin–Elmer Inc., Waltham, MA, USA) in the range of 4000–400 cm^−1^ with a resolution of 4 cm^−1^.

### 4.8. Lactobacillus plantarum *Encapsulation Rate*

*Lactobacillus plantarum* was inoculated in de Man, Rogosa, and Sharpe (MRS) medium and cultured at 37 °C for 16 h. Subsequently, the bacterial suspension was centrifuged at 8000 rpm for 10 min at 4 °C. The cells were washed and suspended twice with 0.85% sterile physiological saline, and then collected, and the bacterial suspension was added to the SA/IN composite solution and mixed evenly. The composite beads carrying bacteria were prepared using the above method. The beads loaded with *Lactobacillus plantarum* were placed in PBS solution and stirred for 30 min, the obtained bacterial suspension was serially diluted, and the viable bacteria count was determined using the plate count method. The formula for calculating encapsulation rate was as follows [37,38].

Encapsulation efficiency (%) = collected viable count/total viable count × 100%(3)

### 4.9. Stability of Lactobacillus plantarum Loaded into SA/IN Beads

The free *Lactobacillus plantarum* and SA/IN beads with 8% IN were placed in 10 mL of sterile physiological saline and stored at 4 °C for 6 days. Samples were taken at 0, 2, 4, and 6 days to determine the viable bacterial count in the system. In the heat treatment experiment, the free *Lactobacillus plantarum* and SA/IN beads were treated and then kept at 37 °C, 45 °C, 50 °C, 55 °C, and 60 °C for 5 min. Subsequently, the glass bottles were immediately placed in an ice bath for 10 min before the viable bacterial count was determined.

### 4.10. Gastrointestinal Activity of Lactobacillus plantarum Loaded into Beads

First, simulated gastric fluid (SGF) and intestinal fluid (SIF) were prepared according to a previous study [39]. For the SGF, pepsin (0.32%) and sodium chloride (0.2%) were mixed, and the pH of the solution was adjusted to 2.0 with 1 M HCl. For SIF, trypsin (0.1%) and porcine bile salt (0.08%) were prepared with 0.2 M PBS solution and mixed well. Finally, SGF and SIF were incubated at 37 °C for 20 min, and then filtrated through a 0.22 μm membrane. *Lactobacillus plantarum*-loaded SA/IN beads (1 g) were added into SGF (9 mL), and the mixture was continuously incubated at 37 °C. After digestion in SGF for 0, 10, 20, 30, 40 min, the SA/IN beads were removed and cracked to measure the bacterial count. After SGF digestion, 10 mL of SIF was added, and the mixture was continuously shaken and mixed at 37 °C. At each incubation period, the solution (0.1 mL) was subjected to bacterial counts using plate counting.

### 4.11. Micromorphology of SA/IN Composite Beads

SA/IN composite beads with or without *Lactobacillus plantarum* were lyophilized using a vacuum freeze-dryer for 48 h. The *Lactobacillus plantarum-*loaded composite beads chosen were completely digested. The beads were sprayed with platinum, and then the morphology was observed using a Hitachi SEM (Regulus 8220, Tokyo, Japan) with an accelerating voltage of 20 kV.

### 4.12. Statistical Analysis

Each experiment was performed in five independent replicates, and the data are expressed as the mean ± standard deviation. Differences were considered significant at *p* < 0.05, and all statistical analyses were conducted with SPSS 25.0 software using analysis of variance and Duncan’s multiple range test.

## Figures and Tables

**Table 1:** Fitted results of Power law model of SA/IN solutions.

IN Concentration (%)	K (Pa⋅S^n^)	n	R^2^
0	2.76965	0.77338	0.962
2	2.56921	0.77655	0.963
4	2.75043	0.77287	0.961
6	2.64498	0.77819	0.951
8	2.99218	0.77401	0.957

Note: K, n, and R^2^ denote the consistency index, flow behavior index, and coefficient, respectively.

## Data Availability

The data presented in this article/the Supplementary Materials are available from the corresponding author on request.

## Supplementary Materials

The following supporting information can be downloaded at https://www.mdpi.com/article/10.3390/gels12070593/s1, Figure S1: The surface roughness analysis of SA/IN composite beads using SEM images.
