# Supplementary material for: Fabrication of Sodium Alginate/Inulin Synbiotic Beads for Protection and Delivery of Lactobacillus plantarum in Storage and Simulated Gastrointestinal Conditions
Source: Gels. 2026 Jul 3;12(7):593. doi: 10.3390/gels12070593 (PMC13408560; doi:10.3390/gels12070593)
Supplement: Supplementary file 1 [file gels-12-00593-s001.zip › gels-4383825-supplementary.pdf]

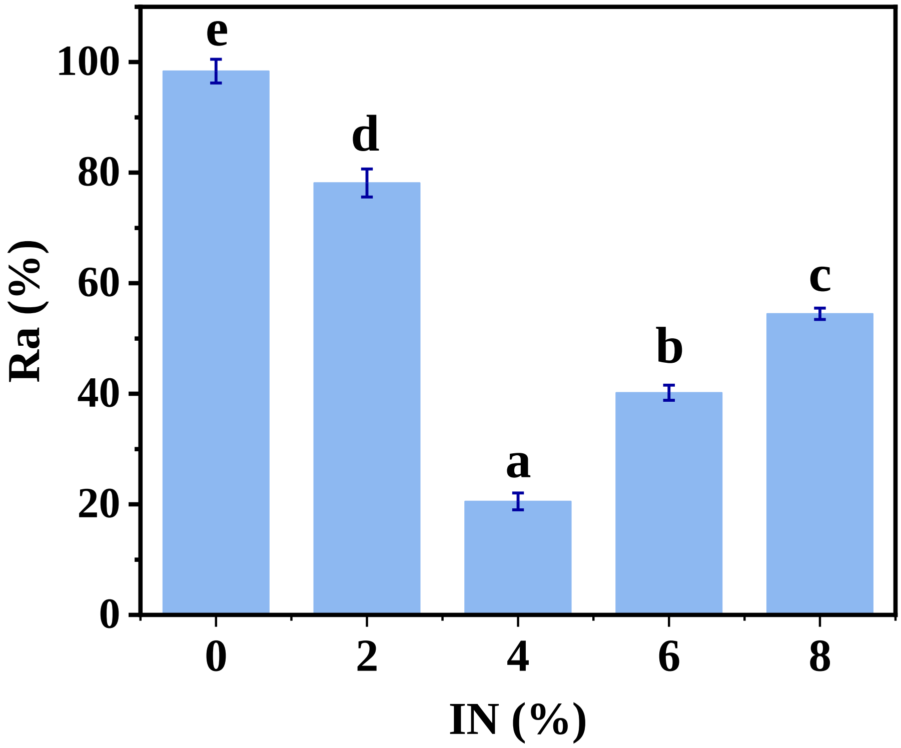

Figure S1 The surface roughness analysis from SEM images of SA/IN composite beads. Different alphabets in the same column demonstrate significant differences ($p < 0.05$).
